# Humans Running in Place on Water at Simulated Reduced Gravity

**DOI:** 10.1371/journal.pone.0037300

**Published:** 2012-07-18

**Authors:** Alberto E. Minetti, Yuri P. Ivanenko, Germana Cappellini, Nadia Dominici, Francesco Lacquaniti

**Affiliations:** 1 Department of Human Physiology, University of Milan, Milan, Italy; 2 Laboratory of Neuromotor Physiology, IRCCS Santa Lucia Foundation, Rome, Italy; 3 Center of Space BioMedicine, University of Rome Tor Vergata, Rome, Italy; 4 Department of Systems Medicine, Neuroscience Section, University of Rome Tor Vergata, Rome, Italy; Universidad Europea de Madrid, Spain

## Abstract

**Background:**

On Earth only a few legged species, such as water strider insects, some aquatic birds and lizards, can run on water. For most other species, including humans, this is precluded by body size and proportions, lack of appropriate appendages, and limited muscle power. However, if gravity is reduced to less than Earth’s gravity, running on water should require less muscle power. Here we use a hydrodynamic model to predict the gravity levels at which humans should be able to run on water. We test these predictions in the laboratory using a reduced gravity simulator.

**Methodology/Principal Findings:**

We adapted a model equation, previously used by Glasheen and McMahon to explain the dynamics of Basilisk lizard, to predict the body mass, stride frequency and gravity necessary for a person to run on water. Progressive body-weight unloading of a person running in place on a wading pool confirmed the theoretical predictions that a person could run on water, at lunar (or lower) gravity levels using relatively small rigid fins. Three-dimensional motion capture of reflective markers on major joint centers showed that humans, similarly to the Basilisk Lizard and to the Western Grebe, keep the head-trunk segment at a nearly constant height, despite the high stride frequency and the intensive locomotor effort. Trunk stabilization at a nearly constant height differentiates running on water from other, more usual human gaits.

**Conclusions/Significance:**

The results showed that a hydrodynamic model of lizards running on water can also be applied to humans, despite the enormous difference in body size and morphology.

## Introduction

**Figure 1:**
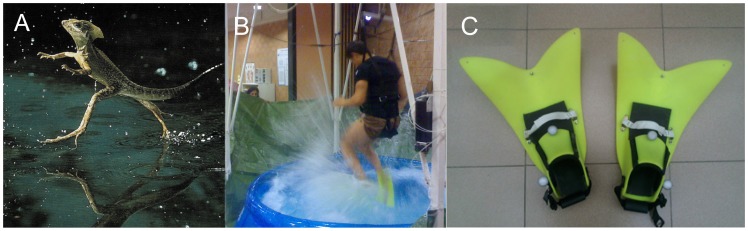
Running on water in Basilisk lizard (A, *Basiliscus basiliscus*), and human in our laboratory conditions (B). The fins used are illustrated in C.

Running on top of a water surface is a task that only few animals can accomplish [1]. In fact, the organisms most successful in this task are water strider insects, which stay afloat by using surface tension to sustain their small body weight [1]. Because the net surface tension force scales with perimeter, but gravity forces scale with volume, surface tension cannot support larger bodies. Bigger animals use a different strategy to avoid sinking while running: they strike the surface with sufficient vigor to generate hydrodynamic forces on their driving legs to support their weight [1]. The Basilisk lizard (*Basiliscus basiliscus*, 90 g, Fig. 1A) has been extensively studied for its ability to run on water by using very fast (8-Hz stride frequency) slaps and strokes [2], [3]. On the heavy side, the Western Grebe (*Aechmophorus occidentalis*, 1.5 kg, Fig. 1B) is a bird capable of a courtship involving running on water for about 20 m at a stride frequency of about 7 Hz.

Notwithstanding various internet hoaxes, humans are apparently incapable of walking or running on water. In their classic study [2] of the Basilisk lizard, Glasheen and McMahon calculate the unsurprising result that humans are far too big and weak to splash their feet hard enough to hold their weight. According to their estimates [2], humans would be able to run on water only if they were able to slap water at speeds >30 m/s, which they estimate would require about 15 times a human’s available muscle power.

However, there are two ways of circumventing these limitations. One way is by reducing gravity, and the other one is by running with flotation devices (giant shoes or fins) as envisaged by Leonardo da Vinci. Bush and Hu [1] calculated that, even at very high slapping speeds of the feet (10 m/s), the area of the feet would need to be about 1 m^2^ in order for a human to walk or run on water in normal gravity.

Here we consider a combination of these two mechanisms: relatively small fins (less than 0.1 m^2^) to increase the water reaction force for a given foot motion, and reducing gravity (to about 20% of Earth gravity) to reduce body weight. To our knowledge, nobody has previously tested the level of gravity at which humans could run on water, nor has anybody tested whether the hydrodynamic model previously developed for lizards by Glasheen and McMahon also applies to humans, despite the enormous difference in body size and morphology. These issues are relevant in the context of comparative physiology. In addition, the reorganization and adaptation of locomotor patterns to the water surface may be of interest for the construction of biologically inspired robots [4], [5], and for searching new forms of the locomotor repertoire [6], [7].

Using both theoretical models and experiments, here we find what combinations of stride frequency, gravity and mass allow a human to run on water like the Basilisk lizards and Western Grebes.

## Results and Discussion

**Figure 2:**
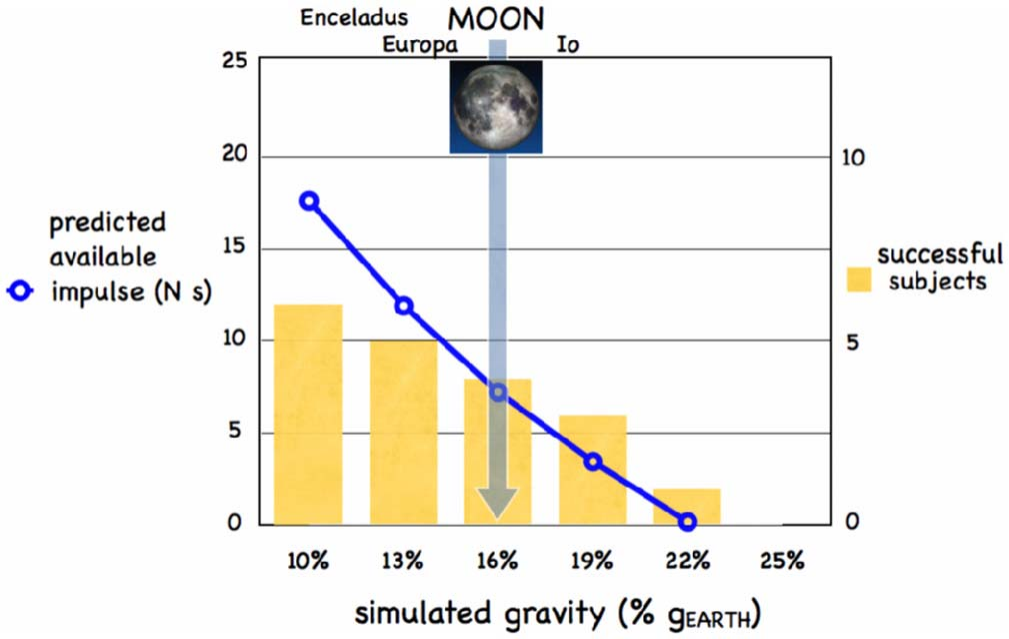
The curve represents the net vertical impulse available ( = 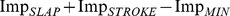), as predicted by the illustrated model. Bars represent the number of subjects, out of 6, capable to avoid sinking at different simulated gravity values. Both variables show that 22% of g_EARTH_ is the maximum gravity at which humans can run on water, when assisted by a small rigid fin.

We experimentally tested whether humans could generate enough muscle power to run in place over a wading pool under simulated reduced gravity (Fig. 1C). Participants, wearing relatively small fins (Fig. 1D) and a harness attached to a constant force unloading system, experienced different levels of simulated gravity (range 10–25% g_EARTH_, see Materials and Methods). The size of the fins, expressed relative to the leg length (i.e. hip-to-heel distance) was roughly comparable to the relative size of the lizards’ feet [8]. For instance, a typical lizard of 10 g mass has a foot size with *r_EFF_*  = 0.008 m and a leg length of 0.035 m [8] (ratio = 0.229). Our human subjects with fins had a similar relationship (*r_EFF_*  = 0.17 m, leg length = 0.8 m, ratio = 0.213). When using *M*  = 66 kg, *r_EFF_*  = 0.17 m, *u_SLAP_*  = 2.504 m/s and *t_PUSH_*  = 0.295 s, the model predicts that it is possible to run on water for 0<*g*≤2.16 m/s^2^, which corresponds to an upper limit of about 0.22 g_EARTH_ (Fig. 2, curve of net available vertical impulse. See Modelling). The two model parameters *u_SLAP_* (slap speed) and *t_PUSH_* (push duration) were derived from the experiments in the wading pool (Table 1). These parameters depend on human physiological constraints which are likely applicable to Earth’s gravity and reduced hypogravity. Interestingly, the model predicted that 82% of the total impulse is contributed by the stroke at 0.22 g_EARTH_, similar to the adult Basilisk lizard [8], [9]. The stroke impulse is further partitioned into 46% and 54% contribution due to hydrodynamic and hydrostatic components of the push, respectively. The model also showed that the maximum body mass compatible with running on water, at the gravity of the Moon (0.16 g_EARTH_) and at a stride frequency of 1.7 Hz, is 73 kg.

**Table 1:** Successful subjects, stride frequency (mean±SD) and maximal knee vertical speed during walking on water at different simulated gravity levels.

gravity	Successful subjects	Stride Frequency	Knee Vertical Speed
(%g_EARTH_)	n	(Hz)	(m/s)
10%	**6**	**1.591**	**−2.067**
		*±0.345*	*±0.279*
13%	**5**	**1.595**	**−2.449**
		*±0.239*	*±0.295*
16%	**4**	**1.638**	**−2.756**
		*±0.264*	*±0.361*
19%	**3**	**1.727**	**−2.808**
		*±0.068*	*±0.588*
22%	**1**	**1.920**	**−2.440**

**Figure 3:**
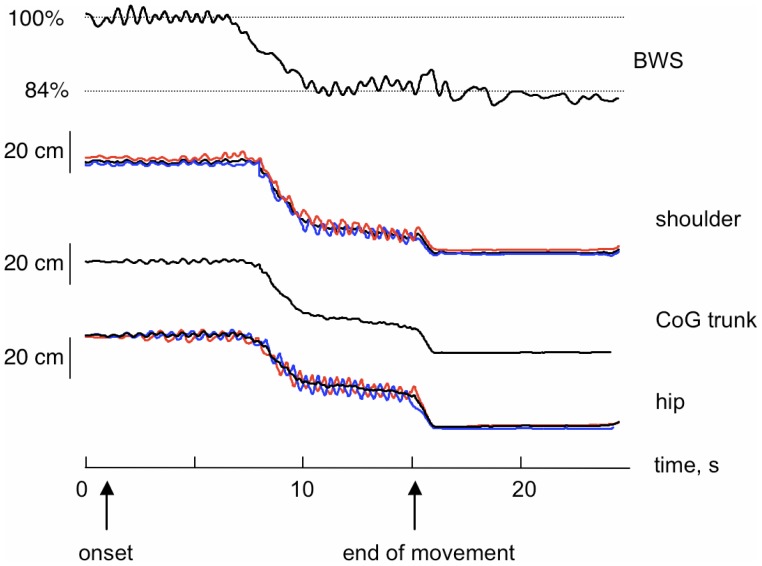
Experimental tracings at 16% g_EARTH_ (Moon) simulated gravity. The subject initially experienced 0% gravity (100% body weight suspension, BWS) then the system was gradually set to the desired value. The upmost signal reflects the force measured by the load cell, while the lower curves represent the vertical coordinate of hip and shoulder, as measured by the motion analysis system. Red and blue curves refer to right and left markers, respectively, while the black one is the average value. ‘CoG trunk’ curve has been calculated as the average of the 4 markers to represent the vertical motion of the head-trunk segment, which approximates the body centre of mass.

**Figure 4:**
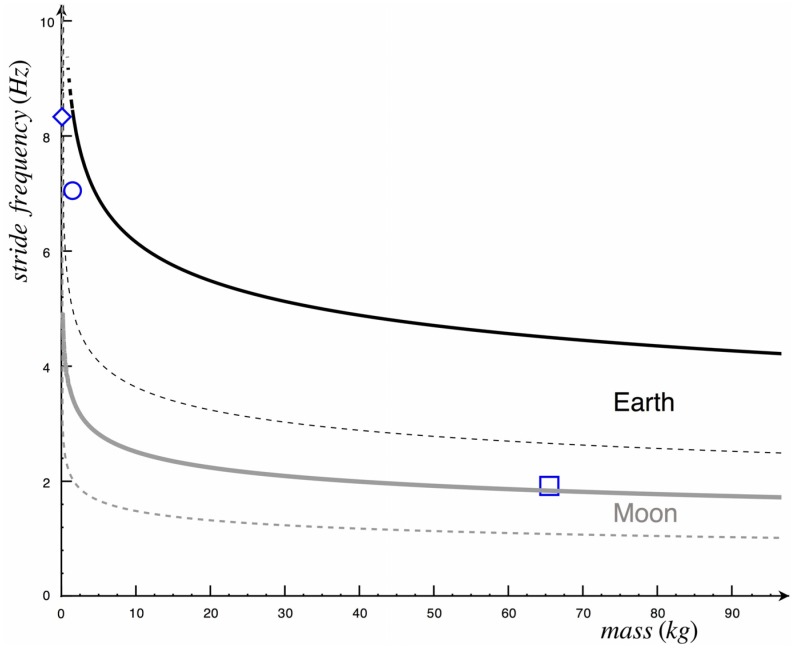
Predictions for two gravity accelerations (Earth - black and Moon - grey) are shown in terms of body mass and stride frequency. The graphic area below the dashed curves represents the mass-frequency combinations at which water cavity seals before protraction and the impulse is not enough to run on water. The graphic area between the dashed and the solid curves represents the mass-frequency combinations at which the impulse is still not enough but the water cavity does not seal before protraction. The graphic area above the solid curves is the ‘safe area’ where mass and frequency involve a sufficient impulse and water does not seal before the end of protraction. Symbols represent the Basilisk lizard (open diamond), the Western Grebe (open circle) and humans (open square).

As predicted by the model, our experiments show that the highest gravity for which a person can run on water is about 0.22 g_EARTH_. All subjects were able to avoid sinking at 10% g_EARTH,_ and a decreasing number of them were successful at higher gravities (Fig. 2, bars). We also found that the subject-chosen stride frequency and maximum vertical speed of the knee were both independent of the gravity level (see Table 1). Figure 3 shows the time-course of the vertical position of the markers located at the hip and shoulder, from which the position of the center of body mass was obtained. Equation 21 produced the curves shown in Figure 4.

One can notice from Fig. 2 that the number of participants who succeeded in running on water at progressively higher gravity levels decreased in parallel with a similar decrease of the net available vertical impulse predicted by the model for these gravity levels. To compute the net vertical impulse, we used values of *M*, *r_EFF_*, *u_SLAP_* and *t_PUSH_* averaged across participants and experiments. Thus, at gravities lower than the 22% g_EARTH_ limit, the available impulse potentially allows almost every subject of normal size to run on water, while only the most skilled and fit subjects can sustain running on water when the net impulse (generated by muscles and gravity, see eq. 4) is close to zero and the effort is almost maximal.

Consider a situation in which a mechanical power of about 888 W is needed by the stroke. The maximum muscle power needed to avoid sinking can be calculated by multiplying the overall drag (eq. 9) by the speed at which the vertical movement occurs (assuming a constant *u* of 2.5 m/s, see Table 1). Alternatively, maximum power can be calculated as body weight multiplied by vertical displacement of the foot and the step frequency: also this calculation provides a value of 888 W. The combined effect of the saturation of muscle power (close to its maximum value) and of the inability to move the legs at higher speeds resulted in a monotonic increment of the foot excursion under the water surface (Table 1), until the subjects tended to sink with increasing level of gravity.

These experiments suffered from various approximations in our reduced gravity simulations. First, although the overall weight is reduced by the lifting cord, both the limbs of the participants and the water were still affected by the full forces of Earth’s gravity. These affect the limb dynamics and the hydrostatic terms (eq. 9), respectively. Moreover, running in place implied that the impulse generated by the muscles was all directed to lift the body vertically, with no power left for the forward thrust needed to progress horizontally. Finally, also as a consequence of running in place, calm water was never available at successive steps.

Water refills the air cavity sooner on Earth than on the Moon (0.30 s and 0.75 s respectively, see t_SEAL_ in eq. 2). Therefore, our participants had to adopt a higher stride frequency, at all simulated gravity levels, than the frequency people would presumably adopt in true hypogravity. Also, the waves generated by each foot-stroke progress at a much slower speed (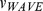, m/s) in true hypogravity [10]:
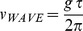
(1)where 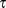 (s) is the wave period. In true hypogravity, the above factors would allow each step to occur on unperturbed water and at a slower frequency, resulting in a higher power and efficiency available from muscles [11] and decreasing the hydrodynamic losses. On the other hand, the hydrostatic component of the impulse is higher during simulated hypogravity. The cancelling of these contrasting effects may have helped the close match we found between theory and experiments.

Previous investigations suggest how gait mechanics are affected by reduced gravity (e.g. [12]), and predict dynamically equivalent speeds for human locomotion on different planets [13]. Here we noticed some striking differences between running on water and normal running on firm Earth ground. Most noticeable is that the upper limbs and body move little (Fig. 1C). Moreover, by inspecting the footage of Basilisk lizards, of Western Grebes and of our subjects, we note that the vertical oscillations are almost nil in both cases (see CoG trunk trace from 10 to 15 s in Figure 3), differentiating this gait from usual terrestrial running (oscillation range of 0.08–0.10 m). Although some upper body markers have vertical motions, there is an out-of-phase pattern of contralateral markers (Fig. 3), as opposed to the in-phase relationship observed in terrestrial running.

For a given duty factor and frequency, the oscillations of the center of mass are constrained by the momentum-balance equations. One extreme condition would involve an impulse once in the middle of each stance phase. The other extreme condition would involve a constant force during the contact phase. Running on water and on land can lead to differences in CoG excursions, only to the extent that these boundary conditions allow. The situation here is closer to the second ‘extreme’, because the alternate ‘cycling’ movement of the two lower limbs probably generates an almost constant force. In lizards and western grebes (see Movies S1, S2 and S3), the head does not move at all vertically. By considering the reciprocal movements of the lower limbs and the compliant water surface, the relative lack of CoG movements is to be expected. Also, the duty factor is close to 0.5 (see above), a value considered as the separator between pendulum-like (walk) and bouncing (run) locomotion paradigms [7], [14]. It is interesting how the bicycle-like style of locomotion (with little changes in the potential energy of the body center of mass) is accompanied by a pedaling (rotating) moving pattern of the lower limbs in both lizards, birds and humans during water running (see Movies S1, S2 and S3). The only limited analogy with terrestrial running is the possible use of pseudo-elastic mechanics to save energy. If t_SEAL_ were long enough (as in true hypogravity), the hydrostatic reaction to the push (which is greater at deeper air/water interfaces) could act as a Hookean spring and assist after the slowing of the limb during the end of the pushing phase.

Regarding the comparison of the present levels of simulated reduced gravity with the gravity of other planets, we note that the limit value of 0.22 g_EARTH_ that we found for water running would include the Moon, four Galilean moons of Jupiter (Io, Ganymede, Callisto, Europa), Saturn moon Enceladus, Pluto, and other 126 celestial objects in the Solar System (Galilean moons and Enceladus revealed traces of water ice or vapor on their surface).

## Materials and Methods

We experimentally tested whether lower limb muscles could generate enough power to run in place over an inflatable wading pool (Fig. 1C). Six participants (mean mass 66 kg and height 1.72 m), wearing two small fins (Pro Force Fins, Bob Evans Designs, USA; surface area of each fin 0.075 m^2^, stiffened along their sagittal plane by an aluminium rod) and a harness attached to a constant weight unloading system [15], experienced 6 different levels of simulated gravity (range 10–25% g_EARTH_). The study was in accordance with the Declaration of Helsinki and written informed consent was obtained from all participants according to procedures approved by the Ethics Committee at the Santa Lucia Foundation. The participant displayed in Figure 1 and the video provided consent for publication.

The body weight support (BWS) was obtained by means of a pneumatic device that applies a controlled upward force at the waist, close to the centre of body mass (WARD system [16]) via a parachute harness (Reha, BONMED, Germany). The BWS mechanism consists of a mechanical gear driven by a pneumatic cylinder, equipped with safety stops. It is held in a cart that slides forwards and backwards over a track formed by a double steel beam, mounted in the middle of the upper side of a parallelepiped steel frame. Very low friction sliding of the mechanism ensures that only vertical forces are applied to the participant. The subject is supported in a harness, pulled upwards by a steel cable connected to the piston of the pneumatic cylinder. Total vertical excursion admitted is 1 m, so that the device, without any regulation, adapts itself to the participant’s height or helps raising him over the surface for air-stepping (100% BWS). WARD exerts the preset unloading force independent of the position of the center of body mass, thus simulating a reduced-gravity environment. A load cell (FGP, type FN3030, France) is positioned in-series with the suspension cable to measure the actual delivered force. The desired unloading force (expressed as percent of subject’s weight) is set on the control computer that accordingly adjusts the pressure inside the pneumatic cylinder. Preset BWS values were applied using a ramp-up (20 N/s, about 30 s to reach 100% BWS), hold (20 to 100 s) and ramp-down (about 30 s) profile of unloading force. The error in the force applied to a subject and the dynamic force fluctuations monitored by the load cell are estimated to be less than 5% of body weight (see Fig. 3, upper trace). The trials were recorded as successful when participants were able to avoid sinking for at least 7–8 seconds (the hypothetical time epoch necessary to cross a small swimming pool).

During the experiments, 3D motion of markers located on main joints was captured by an optoelectronic system (Vicon-612, Oxford Metrics, UK). We recorded kinematic data bilaterally at 100 Hz by means of 9 TV cameras spaced around the wading pool. Infrared reflective markers (diameter 14 and 25 mm) were attached on each side of the participant to the skin overlying the following landmarks: gleno-humeral joint (GH), the midpoint between the anterior and the posterior superior iliac spine (ilium, IL), greater trochanter (GT), lateral femur epicondyle (LE) and lateral malleolus (LM). The spatial accuracy of the system is better than 1 mm (root mean square). LM marker was often lost from tracking due to water splashes.

### Modelling

We follow the approach proposed by Glasheen and McMahon [2], [8], [9] for the lizard, and we extend it to humans running on water at different gravity levels. The general outline of the physical problem and the corresponding equations are similar to those presented by Glasheen and McMahon [2], [8], [9], but the model parameters are adapted to fit humans. A first constraint for running on water is that the air cavity created by each foot-stroke stays open until the end of limb extension. In this way, net propulsion is obtained because, as in rowing, the recovery movement occurs in air, a fluid 800 times less dense than water. Secondly, enough thrust has to be generated to sustain the body weight.

In the following, g_EARTH_ denotes Earth gravity (9.81 m/s^2^), *g* denotes a variable value of gravity acceleration, the pushing surface corresponds to the bottom of the foot or fin, and the radius is the radius of a disk with the same area as the foot or the fin.

For a given *g* value and an effective radius *r_EFF_* of the slapping surface, water refills the cavity in a time *t*_SEAL_ [8], [9]:
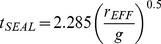
(2)Therefore, the minimum stride frequency f_MIN_ required to run on water is:
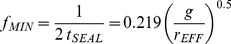
(3)The factor of two is required as seal refers to the closing cavity during one step, while fMIN refers to the stride (2 steps) frequency.

In accord with [8], we set
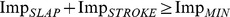
(4)In other words, the net vertical impulse generated by slapping and pushing on water must be greater or equal than the one related to g_EARTH_. Imp_MIN_ refers to the minimum vertical impulse that is needed to keep the body with zero average vertical velocity over a step. Thus, it is a general constraint to avoid sinking into the water.

For a biped (such as man or running lizards), Imp_MIN_ at an arbitrary gravity *g* is
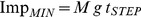
(5)where *M* is the mass of the runner and *t*_STEP_ is the duration of half locomotor cycle.

To avoid sinking:
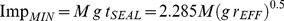
(6)Here we assume that steps occur with a duty factor (the fraction of the stride during which one foot is in contact with water) equal to 0.5. This is the most conservative approach (requires the least average support force). The average support force has to equate body weight (BW). With shorter times (duty factor smaller than 0.5), the average vertical force over the step must be higher than BW, presumably adding muscular effort. For example, with a duty factor of 0.25, the average vertical force during a step would be 2 BW.

In the Glasheen et al model, the forces of water on the fins have three parts. These are the forces that add to make the impulse which counteracts the weight of the person. The first is the slap impulse:
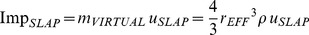
(7)where m_VIRTUAL_ (kg) is the virtual mass of water accelerated during impact, ρ is water density (kg/m^3^) and *u_SLAP_* represents the impact speed (m/s) of the foot [8].

The second component is the stroke impulse:
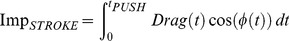
(8)where 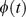 is the time course of changes of orientation of the pushing surface ( = 0 when the surface is horizontal). A dependence on 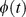 is justified by the fact that, as the foot travels downwards, its orientation changes from horizontal (at the start) to vertical (at the end). The final vertical orientation results in zero contribution to the vertical impulse at the end of the push phase (cos 90° = 0).

The third term is from buoyant forces acting on the bottom of the fin (with no opposing forces at the top), proportional to the mass of the displaced cavity, namely *Sρgh(t),* with *S* as the slap surface area. The *Drag(t)* represents the time course of the force applied to water from the “stroke” term and the buoyant term:
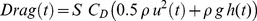
(9)with *C_D_* as the water-entry drag coefficient, and the two terms in brackets referring to hydrodynamic and hydrostatic drag, respectively. The equation can be simplified:
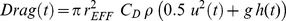
(10)where 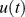 and 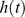 are the instantaneous vertical speed and distance from the water surface of the pushing surface.

As a first approximation, let us assume that during the whole push phase:
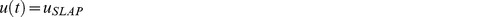
(11)This seems to be a reasonable approximation, because the foot vertical speed tends first to decrease after impact, and then to increase during the following leg extension.

From equation 11
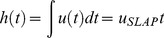
(12)and by computing the definite integral from *t* = 0 to *t* = *t_PUSH_*

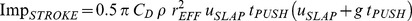
(13)
*CD* is assumed to be equal to 0.703 as in [8].

The problem is to find gravity values for which the constraint equation (Eq. 4) holds, namely.
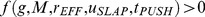
(14)While the parameters *M* and *r_EFF_* are given, the parameters *g*, *u_SLAP_* and *t_PUSH_* must be determined experimentally (see Results).

In order to obtain a more general model that could be applied to animals of different size, we assumed that the body shape of the biped is a vertical cylinder (with mass M, density D, radius R and height H), the base of which is made of two half-circles vertically extending for a distance P, mimicking the feet alternatively pushing against the water. The cylinder shape can be defined by the variable A  =  R/H, and the pushing distance by the variable S  =  P/H. From geometry, it can be calculated that
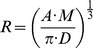
(15)


Also, the effective radius of each of the two pushing half circles, is
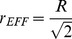
(16)


The slap speed can be calculated as
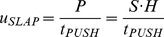
(17)where



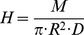
(18)Since
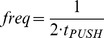
(19)


we obtain
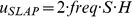
(20)


By assigning the values of A = 0.04 (corresponding to a tall cylinder, a simplification applicable to lizards and humans) and S = 0.50 (corresponding to a leg extension of 50% body length, another reasonable assumption for both lizards and humans) and by assuming a constant body density (D = 1000 kg/m^3^)), we were able to express *r_EFF_, u_SLAP_* and *t_PUSH_* as a function of mass and stride frequency. This allowed simplifying equation 14 into:
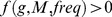
(21)and we could make predictions based on these three variables only. For example, for a given gravity and cylinder mass, equation 21 yields the minimum stride frequency to run on water.

## Supporting Information

Movie S1 **Lizard.mov.** A movie showing how the Basilisk lizard (Basiliscus basiliscus) runs on the water surface.
(MOV)

Movie S2 **16%.mov.** A movie showing one of our subjects running in place on water at a simulated gravity of 1/6 of g_EARTH_ (corresponding to the Moon gravity).
(MOV)
